# Human Cytomegalovirus UL23 Antagonizes the Antiviral Effect of Interferon-γ by Restraining the Expression of Specific IFN-Stimulated Genes

**DOI:** 10.3390/v15041014

**Published:** 2023-04-20

**Authors:** Hankun Wang, Weijian Peng, Jialin Wang, Chunling Zhang, Wangchun Zhao, Yanhong Ran, Xiaoping Yang, Jun Chen, Hongjian Li

**Affiliations:** 1 Department of Biotechnology, College of Life Science and Technology, Jinan University, Guangzhou 510632, China; 2 Guangdong Province Key Laboratory of Pharmacodynamic Constituents of TCM and New Drugs Research, College of Pharmacy, Jinan University, Guangzhou 510632, China; 3 Key Laboratory of Ministry of Education for Viral Pathogenesis & Infection Prevention and Control, Guangzhou 510632, China

**Keywords:** interferon-γ (IFN-γ), human cytomegalovirus, HCMV, UL23, IFN-stimulated genes, ISG

## Abstract

Interferon-γ (IFN-γ) is a critical component of innate immune responses in humans to combat infection by many viruses, including human cytomegalovirus (HCMV). IFN-γ exerts its biological effects by inducing hundreds of IFN-stimulated genes (ISGs). In this study, RNA-seq analyses revealed that HCMV tegument protein UL23 could regulate the expression of many ISGs under IFN-γ treatment or HCMV infection. We further confirmed that among these IFN-γ stimulated genes, individual APOL1 (Apolipoprotein-L1), CMPK2 (Cytidine/uridine monophosphate kinase 2), and LGALS9 (Galectin-9) could inhibit HCMV replication. Moreover, these three proteins exhibited a synergistic effect on HCMV replication. UL23-deficient HCMV mutants induced higher expression of APOL1, CMPK2, and LGALS9, and exhibited lower viral titers in IFN-γ treated cells compared with parental viruses expressing full functional UL23. Thus, UL23 appears to resist the antiviral effect of IFN-γ by downregulating the expression of APOL1, CMPK2, and LGALS9. This study highlights the roles of HCMV UL23 in facilitating viral immune escape from IFN-γ responses by specifically downregulating these ISGs.

## 1. Introduction

Human cytomegalovirus is a ubiquitous pathogen that establishes latent infection and persists in the host, which induces severe diseases in immunocompromised populations [1,2,3,4]. HCMV has the largest genome among herpesviruses, with more than 250 open reading frames (ORFs). Most genes encoded by HCMV are not directly involved in viral replication but assist the virus in regulating its relationship with the host, help the virus escape the host’s immune response, and promote further virus replication and spread [5,6,7].

Interferons-γ (IFN-γ) is a multifunctional cytokine that plays an important role in host immunity against viral infection [8,9,10,11]. IFN-γ induces the transcription of many IFN-stimulated genes, which are involved in antigen presentation and immune regulation, thereby conferring enhanced antiviral effects on stimulated cells [12]. IFN-γ can bind to corresponding cell receptors (IFNGR1/IFNGR2), and thus activate the signal transduction pathway of JAK/STAT in cells. Then, the downstream phosphorylated STAT1 form STAT1 homodimers, which are transported to the nucleus and bind to gamma-activated sequence (GAS) elements to induce transcription of various interferon-stimulated genes (ISGs) [13]. Fundamentally, IFNs act by inducing the expression of immunological and antiviral effector ISGs. Der et al. found that a large number of ISGs are regulated following stimulation with IFN-α, IFN-β, or IFN-γ [14]. Many of these encoded proteins have antiviral effects by directly interfering with biochemical and molecular processes required for viral replication. During the long evolutionary process, HCMV has evolved molecular mechanisms to suppress and escape the interferon response [15,16,17]. For example, HCMV US9 protein was reported to reduce the activities of IFN-β responses by targeting mitochondrial antiviral signaling proteins (MAVS) and stimulators of interferon gene (STING)-mediated signaling pathways [18]; HCMV-encoded glycoproteins US7 and US8 target the TLR3 and TLR4 signaling pathways by promoting the degradation of those TLRs, which results in the overall downregulation of the interferon and antiviral cytokine expression [19]; HCMV protein UL31 inhibits cGAS recognition of viral DNA and contributes to evasion of host antiviral immune responses [20]; HCMV-encoded miR-US25-1-5p could evade innate antiviral immunity by targeting transmembrane glycoprotein CD147 [21]. Previous studies in our laboratory have shown that the HCMV tegument protein UL23 can inhibit the activation of the IFN-γ signaling pathway by targeting human N-myc interactor (Nmi) protein and STAT1 (the signal transducer and activator of transcription 1) [22], and it also can attenuate type I interferon-dependent STAT1 phosphorylation and responses [23]. However, the profile of ISGs regulated by UL23 and their antiviral state remain unclear.

In this study, we analyzed interferon-stimulated genes regulated by HCMV-encoded protein UL23 under the activation of IFN-γ and in the context of HCMV infection. We found for the first time that the IFN-γ-stimulated proteins, APOL1 (Apolipoprotein-L1), CMPK2 (Cytidine/uridine monophosphate kinase 2), and LGALS9 (Galectin-9), can restrain HCMV replication, and UL23 could antagonize the antiviral effect of IFN-γ by specifically downregulating these ISGs.

## 2. Materials and Methods

### 2.1. Virus and Cell Culture

HCMV (Towne-BAC and ΔUL23) expressing GFP generated from Towne-BAC isolate (GenBank: AC146851.1) were kindly provided by Prof. Fenyong Liu (University of California, Berkeley, CA, USA) [24,25]. Human foreskin fibroblasts (HFFs) were obtained from Lonza Inc. (Allendale, NJ, USA), and Lenti × 293T was obtained from the American Type Culture Collection (ATCC, Manassas, VA, USA). All cells were cultivated in Dulbecco’s modified eagle medium (HyClone, Logan, UT, USA) supplemented with 10% fetal bovine serum (Gibco, Grand Island, NY, USA), 1% (*w/v*) penicillin, and streptomycin (Gibco, New York, NY, USA) at 37 °C under 5% CO_2_.

### 2.2. Reagents and Antibodies

Recombinant human IFN-γ (300-02) was purchased from PeproTech (Cranbury, NJ, USA). Rabbit anti-FLAG (14793S) and mouse anti-HA (2367S) were obtained from Cell Signaling Technology (Danvers, MA, USA). Mouse anti-β-actin (66031-1-Ig), mouse anti-APOL1 (66124-1-Ig), and mouse anti-LAGSAL9 (17938-1-AP) antibodies were purchased from Proteintech (Wuhan, China), and mouse anti-CMPK2 (PSC-7063-C020) was purchased from ENZO (New York, NY, USA).

### 2.3. RNA-Seq and Data Analysis

RNA libraries were prepared according to the VAHTS mRNA-seq v.2 Library Prep Kit (Vazyme, Nanjing, China), and crude sequencing fragments were generated on an Illumina HiSeq X Ten sequencer. Clean reads of at least 50 nucleotides in length were matched to a transcriptional reference library (Human_hg19_refMrna20 150317as) using the FANSe3 high-precision algorithm. Gene expression levels were quantified in RPKM units.

### 2.4. Virus Infection and Growth Analysis

All HCMVs were handled at a biosafety level 2 (BSL2) containment using the appropriate protocols and practices. HFF cells were seeded at 2 × 10^5^ cells per well in 6-well plates. In triplicate, HFF cells were infected with mock or HCMV (MOI = 1) in an inoculum of DMEM supplemented with 1% fetal bovine serum. The inoculum was replaced with DMEM supplemented with 10% fetal bovine serum after 2 h of incubation with the cells.

The cells and medium were harvested 7 days post-infection to quantify viral growth, and viral stocks were prepared. Viral stocks were serial diluted and used to infect 1 × 10^5^ human foreskin fibroblasts, followed by agar overlay. Viral titer was determined by counting the number of plaques 10–14 days after infection.

### 2.5. RNA or DNA Isolation and Quantitative Reverse Transcription-PCR (RT-qPCR)

Total RNA was extracted from cells with Trizol (Invitrogen, Carlsbad, CA, USA) according to the manufacturer’s manual. RNA (500 ng) was reverse transcribed using a cDNA synthesis kit (TAKARA, Shanghai, China). For HCMV genomic DNA, total DNA was extracted using PureLink Genomic DNA Mini Kit (Thermo Fisher Scientific, Waltham, MA, USA). PCR reactions were performed using SYBR Green Supermix (Applied Biosystems, Foster City, CA, USA). The oligonucleotide primers used were as follows: APOL1 forward (5′-TTCGAATTCCTCGGTATATCTTG-3′), APOL1 reverse (5′-CACCTCCAGTTATGCGTCTG-3′); CMPK2 forward (5′-TCACCTGGTTCAACCTACTCCCT-3′), CMPK2 reverse (5′-ACAGTGGATCTTGGAGTGGGA-3′); LGALS9 forward (5′-TCCACCTGAACCCCCGTTTT-3′), LGALS9 reverse (5′-GACGAAGGGCATT TTTCGGG-3′); IE1 forward (5′-GAAGGTGAAGGTCGGAGTC-3′), IE1 reverse (5′-AAGATGGTGATGGGATTTC-3′); UL83 forward (5′-CCGACAACGAAATCCACAAT-3′), UL83 reverse (5′-TTCTGACCCTGAACCGTAGC-3′); and GAPDH forward (5′-GAAGGTGAAGGTCGGAGTC-3′), GAPDH reverse (5′-AAGATGGTGATGGGATTTC-3′). Thermal cycling conditions were as follows: 50 °C for 2 min, 95 °C for 15 min, and 45 cycles of 95 °C for 30 sec and 60 °C for 1 min. Relative quantitation was determined using the comparative CT method with data normalized to GAPDH. Data analyses were performed using the 2^−ΔΔCt^ method.

### 2.6. Western Blotting Assay

Cell lysates were obtained after incubating cells with RIPA lysis buffer (Sigma Aldrich, St. Louis, MO, USA) supplemented with a protease inhibitor cocktail (Roche, Basel, Switzerland). The protein content was determined by Bradford assay (Bio-Rad, Hercules, CA, USA). Equivalent amounts of protein were separated by SDS-PAGE, transferred onto membranes, reacted with antibodies, stained using a Western chemiluminescent substrate kit (Thermo Fisher, Waltham, MA, USA), and quantitated with a STORM840 PhosphorImager or a gel documentation station (BioRad, Hercules, CA, USA).

### 2.7. RNA Interference

Double-stranded oligonucleotides corresponding to the target sequences were cloned into the pLKO.1 plasmid. The following sequences were targeted for APOL1, CMPK2, and LGALS9: shAPOL1 forward (5′-CCGGCTACTCCTGCTGACTGATAATCTCGAGATTATCAGTCAGCAGGAGTAGTTTTTG-3′), shAPOL1 reverse (5′-AATTCAAAAACTACTCCTGCTGACTGATAATCTCGAGATTATCAGTCAGCAGGAGTAG-3′); shCMPK2 forward (5′-CCGGTTGAGGCCAACAGTGTGTTTCCTCGAGGAAACACACTGTTGGCCTCAATTTTTTG-3′), shCMPK2 reverse (5′-AATTCAAAAATTGAGGCCAACAGTGTGTTTCCTCGAGGAAACACACTGTTGGCCTCAA-3′); and shLGALS9 forward (5′-CCGGCCCACCATCAACAGACTGGAACTCGAGTTCCAGTCTGTTGATGGTGGGTTTTTG-3′), shLGALS9 reverse (5′-AATTCAAAAACCCACCATCAACAGACTGGAACTCGAGTTCCAGTCTGTTGATGGTGGG-3′).

### 2.8. Stable Cell Lines

To generate the stable HFF-C and HFF-UL23-HA lines, PCDH or PCDH-UL23-HA and psPAX2 packaging plasmid, Pmd2-G envelope plasmid were transfected into Lenti × 293T using StarFect High-efficiency Transfection Reagent (GenStar, Beijing, China) according to the manufacturer’s instructions. After 48 h and 72 h transfection, the lentiviral particles were collected, clarified through an 0.45 µM filter, and stored at −80 °C. At this point, the lentiviral particles were added to HFF cells. After 24 h infection, the media was removed, and the HFF cells were cultured in complete media, as described above. The infected HFF cells were selected with puromycin (2 µg/mL).

To overexpress or silence APOL1, CMPK2, and LGALS9, Lenti × 293T were transfected with two packaging plasmids (psPAX2, Pmd2.G) together with a pLenti-LUC-3 × Flag-P2A-C-tRFP or pLenti-APOL1-3 × Flag-P2A-C-tRFP, pLenti-CMPK2-3 × Flag-P2A-C-tRFP, pLenti-LGALS9-3 × Flag-P2A-C-tRFP, shAPOL1, shCMPK2, and shLGALS9 plasmid using StarFect High-efficiency Transfection Reagent (GenStar, Beijing, China) as previously described. The lentiviral particles were collected and then infected with 2 × 10^5^ HFF cells per well. After 48 h, cells were incubated with new media to perform additional experiments.

### 2.9. Statistical Analysis

Each assay was independently performed thrice. All results are given as mean ± SD and analyzed using statistical tools implemented in Prism (GraphPad Prism 8.0). For two samples, an unpaired, two-tailed Student’s *t*-test was used for statistical analysis and the F-test was performed to confirm that the two populations have the same variances. The Shapiro–Wilk normality test was performed to confirm the normal distribution of all datasets. For multiple comparisons, one-way ANOVA was performed followed by a post hoc test. Differences with *p* < 0.05 were considered to be significant.

## 3. Results

### 3.1. RNA-Seq Analysis Revealed That HCMV-Encoded UL23 Protein Inhibited the Expression of IFN-Inducible Genes APOL1, CMPK2, and LGALS9

**Figure 1:**
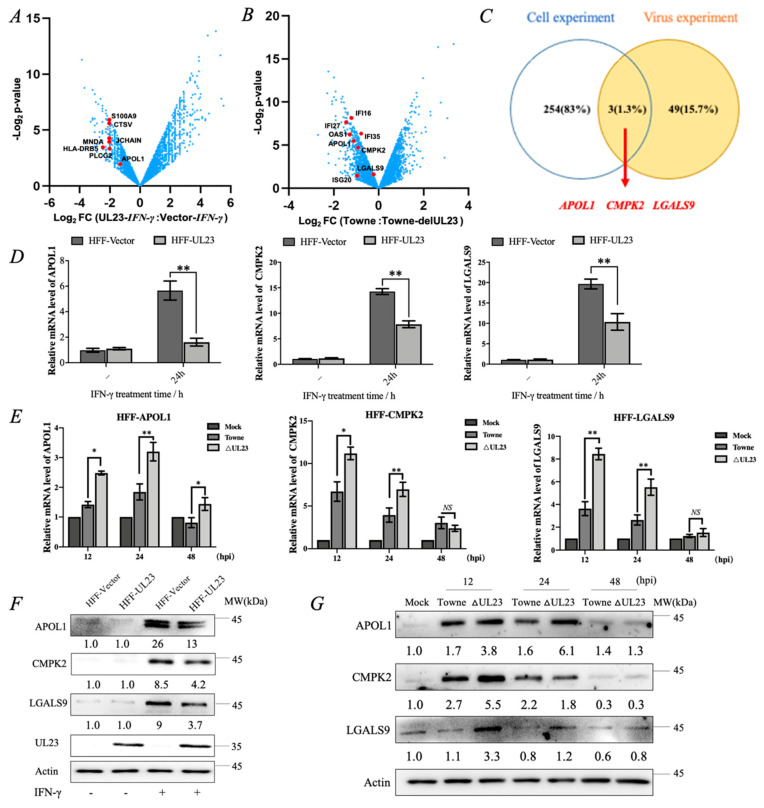
HCMV-encoded protein UL23 inhibits the expression of IFN-inducible genes Apol1, Cmpk2, and Lgals9. (**A**) UL23 stably expressed HFF cells (HFF-UL23) and control cells (HFF-Vec) were treated with 1000 U/mL IFN-γ for 24 h. Volcanic map showing expression of cellular targets of UL23 in HFF cells after stimulation by IFN-γ. (**B**) HFF cells infected with HCMV-Towne or HCMV-Towne-ΔUL23 (MOI = 1) for 24 h. Volcanic map showing the effects of UL23 on host gene expression after HCMV infection of HFF cells. (**C**) Boolean operation analysis showed that HCMV-Towne vs. HCMV-Towne-ΔUL23 differentially expressed genes and Vec-IFN-γ vs. UL23-IFN-γ differentially expressed genes were composed of APOL1, CMPK2, and LGALS9. (**D**) The effects of ectopic expression of UL23 on the gene expression of APOL1, CMPK2, and LGALS9 with or without IFN-γ (1000 U/mL) stimulation. (**E**) HCMV or HCMV-ΔUL23 (MOI = 1) was used to infect HFF cells for different time points. The APOL1, CMPK2, and LGALS9 mRNA expressions were measured through RT-qPCR, with GAPDH as the reference. (**F**) HFF-UL23 and HFF-Vector cells were treated with IFN-γ (1000 U/mL) for 24 h before Western blotting. (**G**) HCMV-Towne or HCMV-Towne-ΔUL23 (MOI = 1) was used to infect HFF cells for different time points, and total cell lysates were analyzed by immunoblot using the indicated antibodies. Each assay was conducted thrice. Data are shown as mean ± SD. * *p* < 0.05, ** *p* < 0.01, NS denotes not significant (*p* > 0.05). Data are representative of three independent experiments with similar results.

Our previous study found that the UL23 protein could inhibit the phosphorylation of STAT1, a core molecule of the IFN-γ signal transduction pathway, thereby increasing the resistance of the HCMV to IFN-γ [22]. However, it is unclear which target genes downstream of the IFN-γ signaling pathway are regulated by the UL23 protein. In this experiment, two cell lines, HFF-Vector (HFF-Vec) and HFF-UL23 (UL23 stable expression cell line), were constructed, and RNA-Seq technology was used to demonstrate the effect of UL23 on ISGs expression after 24 h of IFN-γ stimulation. Analyses revealed 229 downregulated and 28 upregulated differentially expressed genes shared among cells expressing UL23 compared to the control alone (RPKM ≥ 10, Fold change ≥ 1.5 (upregulate) and Fold change ≤ 0.7 (downregulate)). Then, KEGG pathway enrichment and GO analysis were performed on the screened differential genes using DAVID 6.8. The results showed that under the action of IFN-γ, the UL23 protein encoded by HCMV mainly affected the host chemokine signaling pathway, NF-κB signaling pathway, MAPK signaling pathway, and the expression of functionally related genes of innate immunity and inflammatory response (Figure 1A and Figure S1A). It has been reported that HCMV can also induce IFN-γ production when infecting non-immune cells [26]. Next, we comparatively analyzed host genes regulated by UL23 during the HCMV infection and found that a total of 52 differentially expressed genes (38 upregulated and 14 downregulated genes) were screened between HCMV-Towne and HCMV-Towne-∆UL23 (RPKM ≥ 10, Fold change ≥ 2). The analysis found that UL23 mainly regulated the host’s innate immune response during HCMV infection (Figure 1B and Figure S1B).

Next, we found that both differentially expressed genes of HCMV-Towne vs. HCMV-Towne-∆UL23 and HFF-Vec-IFN-γ vs. HFF-UL23-IFN-γ contained APOL1, CMPK2, and LGALS9 (Figure 1C). To validate the transcriptomic data, we performed quantitative real-time PCR (RT-qPCR) and immunoblot to analyze the effect of ectopically expressed UL23 on the expression of APOL1, CMPK2, and LGALS9 upon IFN-γ stimulation or HCMV infection. As expected, UL23 significantly restricted IFN-γ-induced gene expressions of APOL1, CMPK2, and LGALS9 (Figure 1D,F); UL23 also significantly reduced their expression during HCMV infection (Figure 1E,G). These results indicated that HCMV UL23 could restrain the enhancing expression of interferon-γ-inducible genes APOL1, CMPK2, and LGALS9.

### 3.2. IFN-γ-Inducible Genes APOL1, CMPK2, and LGALS9 Inhibit HCMV Replication

**Figure 2:**
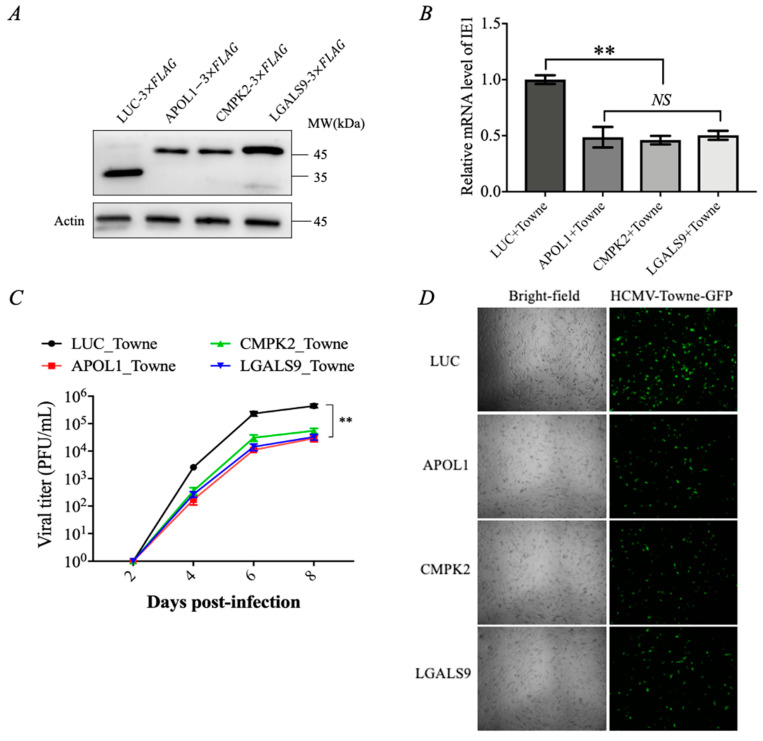
IFN-inducible proteins APOL1, CMPK2, and LGALS9 inhibit HCMV replication. (**A**) HFF cells were transfected with the expression constructs of LUC or APOL1, CMPK2, and LGALS9, and protein levels were analyzed by immunoblot. (**B**) Control and three protein-overexpressing (APOL1, CMPK2, and LGALS9) HFF cells were infected with HCMV-Towne at an MOI of 1. Total RNAs were prepared at 24 h, and the level of IE1 transcripts was measured by RT-qPCR. (**C**) Cell culture supernatants from cells infected with HCMV-Towne at an MOI of 1 were analyzed at different time points for progeny virus titers using infectious center assays in HFF cells. (**D**) HFF cells were infected with HCMV-Towne harboring GFP as a reporter. Cells infected at an MOI of 1 were monitored for the spread of the GFP signal. GFP images merged with phase-contrast images were collected at 7 days post-infection. Scale bar, 50 μm. Data are shown as mean ± SD. ** *p* < 0.01. Data are representative of three independent experiments with similar results.

Based on the reported antiviral functions of these three genes to other viruses [27,28,29], we characterized the roles of APOL1, CMPK2, and LGALS9 in the process of HCMV infection in the following studies. We then examined the effect of exogenous overexpression of APOL1, CMPK2, and LGALS9 on HCMV-Towne replication by constructing lentiviral packaging vectors (Figure 2A). It was found that overexpression of APOL1, CMPK2, and LGALS9 in HFF cells significantly suppressed the expression level of the HCMV-Towne IE1 gene and viral titers compared to control cells (Figure 2B,C). We also found that the effect of these three proteins on HCMV replication was not due to the influence of the levels of the viral genome getting to the nucleus (Figure S2). Cells were infected with HCMV-Towne expressing a GFP reporter and monitored for viral spread. We also observed a low spread of GFP signal in cells for APOL1, CMPK2, and LGALS9 compared with control cells during infections at an MOI of 1 (Figure 2D). These data indicate that the interferon-γ-inducible genes APOL1, CMPK2, and LGALS9 can inhibit HCMV replication.

### 3.3. The Combination of APOL1, CMPK2, and LGALS9 Exhibits Synergistic Anti-HCMV Effects

**Figure 3:**
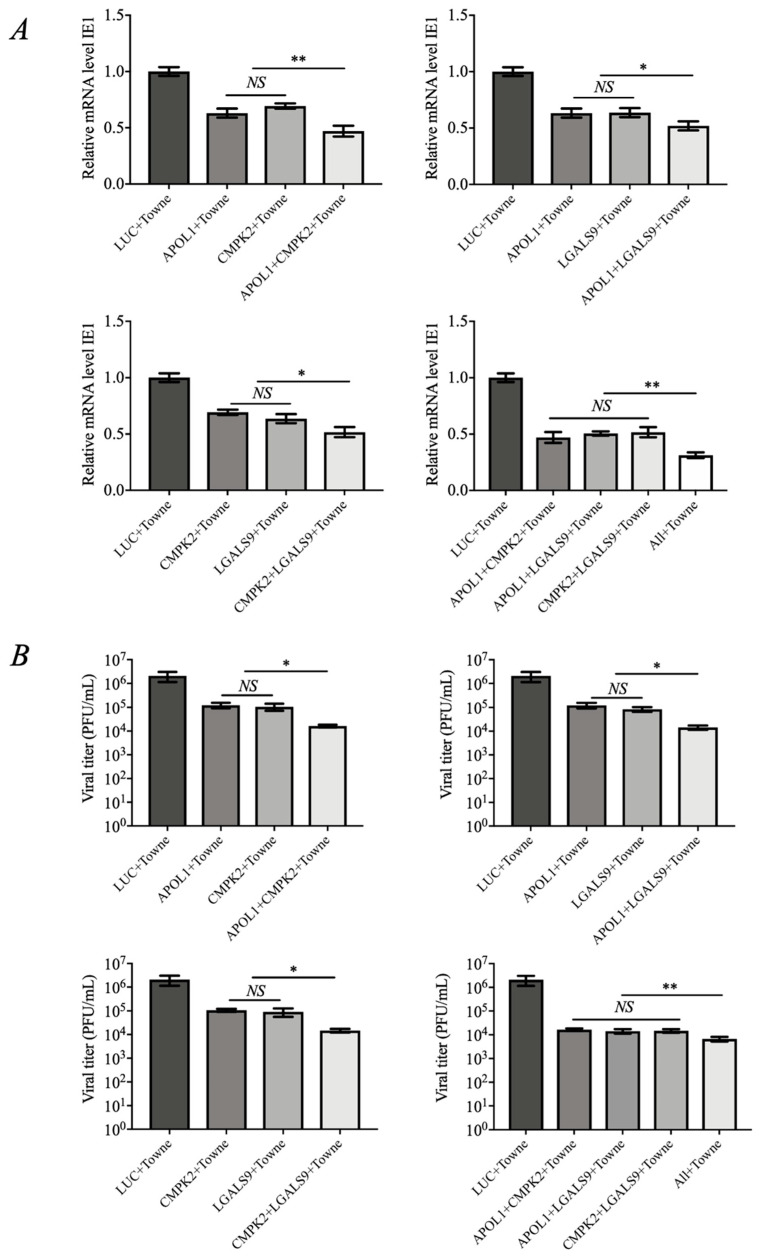
A combination of APOL1, CMPK2, and LGALS9 exhibits synergistic anti-HCMV effects. (**A**) RT-qPCR detection of the synergistic effects of APOL1, CMPK2, and LGALS9 on the expression of the HCMV-Towne IE1 gene (24 h). (**B**) Virus titer determination APOL1, CMPK2, and LGALS9 synergistic effects on HCMV-Towne replication (7 d). Data are shown as mean ± SD. * *p* < 0.05, ** *p* < 0.01, NS denotes not significant (*p* > 0.05). Data are representative of three independent experiments with similar results.

To better elucidate the effects of APOL1, CMPK2, and LGALS9 on HCMV-Towne replication, we further investigated whether APOL1, CMPK2, and LGALS9 synergistically or additively inhibit HCMV-Towne replication in the following experiments. We overexpressed different combinations of these proteins in HFF cells. The results showed that compared with the control group (LUC), combinational overexpression of the two or three proteins among APOL1, CMPK2, and LGALS9 could significantly inhibit the expression level of the viral IE1 gene and the replication of progeny viruses (Figure 3A). More importantly, multiple combinations more significantly inhibited the IE1 gene expression and progeny virus replication than single-expressed proteins in cells (Figure 3B). The above results suggest that APOL1, CMPK2, and LGALS9 could act additively or even synergistically to inhibit HCMV replication.

### 3.4. UL23 Limited the Antiviral Effect of IFN-γ by Downregulating the Expression of APOL1, CMPK2, and LGALS9

**Figure 4:**
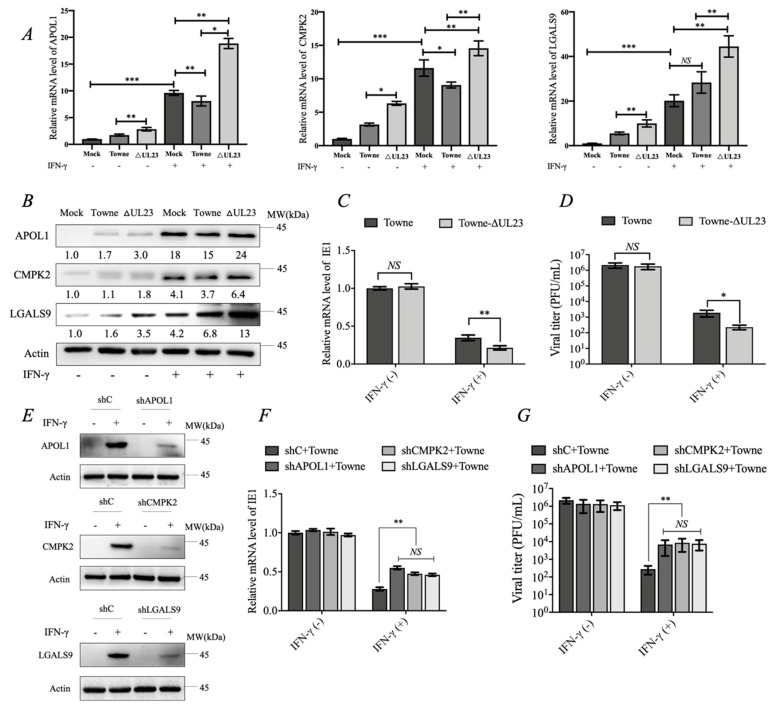
UL23 resisted the antiviral effect of IFN-γ by downregulating the expression of APOL1, CMPK2, and LGALS9. (**A**,**B**) The effect of HCMV UL23 on the gene expression of APOL1, CMPK2, and LGALS9 with or without IFN-γ (1000 U/mL) stimulation during the HCMV infection (before HCMV infection, HFF cells were pretreated with IFN-γ for 12 h). Total RNAs were prepared at 24 h, the level of genes was measured by RT-qPCR, and total cell lysates were prepared and analyzed by immunoblotting. (**C**) HFF cells were infected with HCMV-Towne or HCMV-Towne-ΔUL23 at an MOI of 1 and treated with or without 1000 U/mL IFN-γ for 24 h (before HCMV infection, HFF cells were pretreated with IFN-γ for 12 h). Total RNAs were prepared at 24 h, and the level of IE1 transcripts was measured by RT-qPCR. (**D**) Cell culture supernatants from cells infected with HCMV-Towne or HCMV-Towne-ΔUL23 at an MOI of 1 were analyzed 7 days post-infection for progeny virus titers using infectious center assays in HFF cells. (**E**) HFF cells were stably transduced to the target gene with lentiviruses containing non-target control shRNA (NT) or shRNAs (APOL1, CMPK2, and LGALS9). (**F**) Control and stable knockdown HFF cells (APOL1, CMPK2, and LGALS9) were infected with HCMV-Towne at an MOI of 1 and treated with or without 1000 U/mL IFN-γ for 24 h (before HCMV infection, HFF cells were pretreated with IFN-γ for 12 h). Total RNAs were prepared at 24 h, and the level of IE1 transcripts was measured by RT-qPCR. (**G**) Cell culture supernatants from cells infected with HCMV-Towne at an MOI of 1 were analyzed 7 days post-infection for progeny virus titers using infectious center assays in HFF cells. Data are shown as mean ± SD. * *p* < 0.05, ** *p* < 0.01, *** *p* < 0.005, NS denotes not significant (*p* > 0.05). Data are representative of three independent experiments with similar results.

The results of the previous part show that APOL1, CMPK2, and LGALS9 can inhibit HCMV replication. We speculate that the HCMV UL23 protein may reduce the expression of IFN-γ-induced proteins APOL1, CMPK2, and LGALS9 to a certain extent, thereby helping the HCMV to counteract the inhibitory effect of IFN-γ. As expected, the IFN-γ-induced ISGs mRNA and protein expression of APOL1, CMPK2, and LGALS9 are low in most normal cells but highly expressed under the stimulation of interferon-γ. Compared with HCMV-Towne-ΔUL23, the enhancing levels of ISGs were significantly restrained in HCMV-Towne-infected HFF cells with or without IFN-γ stimulation (Figure 4A,B). It can also be seen that IFN-γ can significantly inhibit HCMV replication as previously reported [30]. In the meantime, with IFN-γ treatment, the repression of IE1 mRNA expression and viral titer in the HCMV-Towne group were significantly decreased within HFF cells relative to the HCMV-Towne-ΔUL23 group, suggesting that UL23 exerts a vital part in suppressing the antiviral function of IFN-γ (Figure 4C,D). Notably, our results showed that without IFN-γ treatment, the IE1 mRNA expression and viral titer in HFF cells were not significantly different between HCMV-Towne and HCMV-Towne-ΔUL23 (Figure 4C,D), which may be due to the low expression levels of these three proteins in the host during HCMV infection, which cannot effectively suppress the replication of HCMV. To further investigate the effect of endogenous APOL1, CMPK2, and LGALS9 on HCMV replication, we used shRNA to inhibit the expression of APOL1, CMPK2, and LGALS9 in IFN-γ-stimulated HFF cells (Figure 4E). Under the effect of IFN-γ, silencing APOL1, CMPK2, and LGALS9 expression significantly increased the expression level of the HCMV-Towne IE1 gene and the titer of progeny virus compared with control cells (shC) (Figure 4F,G). These results indicated that the HCMV UL23 protein might limit the antiviral effect of IFN-γ by downregulating the expression of IFN-γ-stimulated genes APOL1, CMPK2, and LGALS9.

## 4. Discussion

HCMV exploits an impressive arsenal of immune evasion genes to establish lifelong persistence in its host, a paradigm of viral immune evasion [7,20,31]. The IFN response is an important part of the human immune response to HCMV infection. Studies have shown that HCMV can encode various viral proteins to inhibit or prevent the host’s IFN response [8,32]. Despite the numerous and multi-faceted antiviral effects of IFNs, HCMV can invade, multiply, and establish persistent infection in healthy hosts. This capacity is a direct result of the strategies employed by HCMV to block IFN-dependent functions, impair IFN induction, and manipulate the physiological outcomes of ISG proteins [33]. Previous studies in our laboratory have shown that the HCMV-encoded UL23 protein can inhibit STAT1 phosphorylation and nuclear translocation, thereby interfering with type I or II IFN signaling, bypassing the antiviral effects of downstream ISGs [22,23]. However, whether UL23 regulates ISGs remain unknown.

Through signaling on certain cell surfaces, IFN ultimately mediates the expression of multiple ISGs, some of which have been found to play important roles in innate immunity against viral infections [12,34,35,36]. Moreover, studies have demonstrated the vital role of some ISGs in restricting the intracellular replication of viruses and bacteria by directly targeting pathways and stages of the pathogen life cycle [32,37,38]. We thus performed RNA-Seq technology to analyze and compare the effect of UL23 on host gene expression under IFN-γ-stimulation or HCMV infection. It was found that the specific host genes regulated by UL23 collectively included Apol1, Cmpk2, and Lgals9, and UL23 could significantly decrease the expression of these interferon-inducible genes (Figure 1). Thus, our work enriches the detailed knowledge of IFN-γ-induced gene expression regulation by UL23.

The complex interaction between HCMV and its host is essential for the outcome of infection. Understanding the mechanisms by which HCMV manipulates ISGs would give an insight into how HCMV establishes lifelong infections and how ISGs play a role in immune defense. As an effective antiviral agent, ISGs can affect virus replication through a variety of mechanisms. For example, 1. inhibition of virus entry; 2. inhibition of virus translation and replication; and 3. inhibitors of viral egress [36]. At present, there are few reports on the molecular mechanism by which ISGs inhibit HCMV replication. APOL1 and CMPK2 are mainly induced by interferon and proinflammatory cytokines, and LGALS9 is widely expressed in cells and immune system tissues and plays a major role in the inflammatory response and immune response against infection. Thus far, APOL1 [27], CMPK2 [28,39], and LGALS9 [29,40] have been reported to inhibit the replication of numerous viruses, including HIV, HBV, dengue virus, and other viruses, and, meanwhile, help the host to resist and mitigate the negative effects of the pathogens [41,42,43], but their biological functions remained enigmatic to HCMV. It has been reported that the addition of lgals9 protein to the culture medium can prevent HCMV infection by affecting the fusion of HCMV with the cell membrane [44]. However, the effect of the other two proteins on HCMV replication has not been reported yet. Our study showed that endogenous expression of these three proteins could inhibit HCMV replication but didn’t influence the levels of viral genome entering into the nucleus (Figure 2). However, a better mechanistic understanding of individual ISGs could lead to the development of new treatments. Therefore, more experimental evidence is needed to confirm how these interferon-induced proteins inhibit HCMV replication.

A recent study revealed the complex network that ISGs create during viral infection, adding even greater complexity to the host’s immune response against infection [45]. Most ISGs do not inhibit virus infection when expressed individually. It is clear that the IFN system did not evolve to have one potent virus-specific ISG per virus, but rather to work in a combinatorial fashion [36]. Given that all three proteins are regulated by UL23, we expected that when combined they would have additive and/or synergistic effects. We found that APOL1, CMPK2, and LGALS9 cooperated to decrease HCMV-Towne replication (Figure 3). In follow-up studies, we found that UL23 could counteract the antiviral effect of IFN-γ by downregulating the expression levels of IFN-γ-stimulated genes APOL1, CMPK2, and LGALS9 (Figure 4). This result provides new insights into the understanding of the immune evasion strategy of HCMV.

## 5. Conclusions

**Figure 5:**
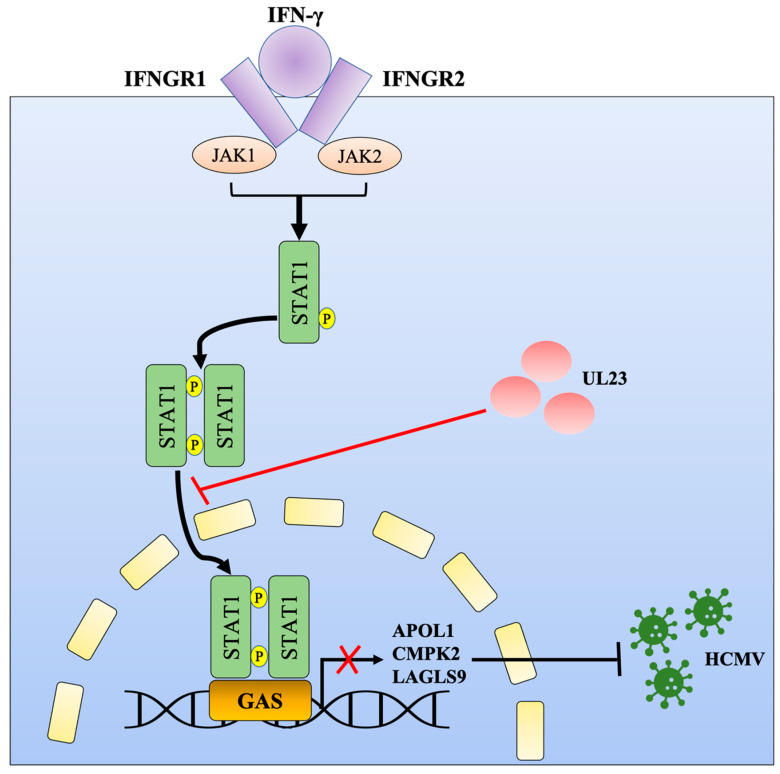
A model for the HCMV UL23-mediated suppression of IFN-γ-mediated ISG transcription (APOL1, CMPK2, and LGALS9). During HCMV infection, UL23 disrupts the phosphorylation and nuclear localization of STAT1 and reduces ISG transcription and GAS promoter activation; in the meanwhile, it restrains the inhibitory effects of APOL1, CMPK2, and LGALS9 on HCMV replication.

In summary, our study shows that the antiviral effect of HCMV UL23 on IFN-γ is not only limited to the inhibition of IFN-γ signaling reported previously [22] but also restrains the specific antiviral ISGs expression (Figure 5). Therefore, a complete understanding of the role of UL23 in IFN-γ responses would contribute to our understanding of how HCMV regulates IFN-γ responses to evade intrinsic and innate defense mechanisms.

## Data Availability

All the data generated during the current study are included in the manuscript.

## Supplementary Materials

The following supporting information can be downloaded at: https://www.mdpi.com/article/10.3390/v15041014/s1, Figure S1: KEGG pathway enrichment and GO analysis of the differential genes regulated by UL23; Figure S2: The effect of APOL1, CMPK2, and LGALS9 on HCMV entry into cells.
