# Supplementary material for: Human Cytomegalovirus UL23 Antagonizes the Antiviral Effect of Interferon-γ by Restraining the Expression of Specific IFN-Stimulated Genes
Source: Viruses. 2023 Apr 20;15(4):1014. doi: 10.3390/v15041014 (PMC10145438; doi:10.3390/v15041014)
Supplement: Supplementary file 1 [file viruses-15-01014-s001.zip › viruses-2300139-supplementary.pdf]

A

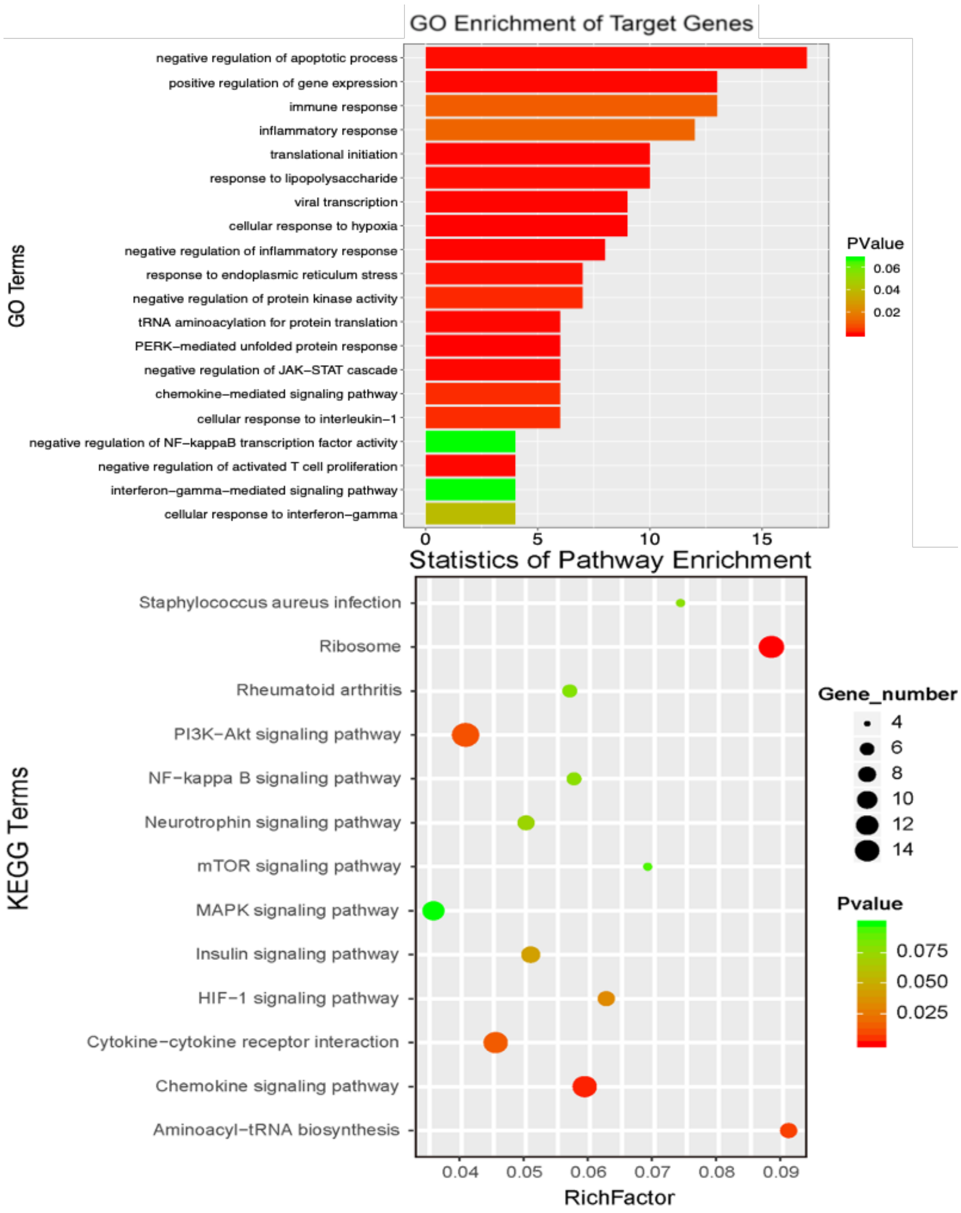
GO Enrichment of Target Genes
negative regulation of apoptotic process
positive regulation of gene expression
immune response
inflammatory response
translational initiation
response to lipopolysaccharide
viral transcription
cellular response to hypoxia
negative regulation of inflammatory response
response to endoplasmic reticulum stress
negative regulation of protein kinase activity
tRNA aminoacylation for protein translation
PERK−mediated unfolded protein response
negative regulation of JAK−STAT cascade
chemokine−mediated signaling pathway
cellular response to interleukin−1
negative regulation of NF−kappaB transcription factor activity
negative regulation of activated T cell proliferation
interferon−gamma−mediated signaling pathway
cellular response to interferon−gamma
GO Terms
0
5
10
15
PValue
0.06
0.04
0.02
Statistics of Pathway Enrichment
Staphylococcus aureus infection
Ribosome
Rheumatoid arthritis
PI3K−Akt signaling pathway
NF−kappa B signaling pathway
Neurotrophin signaling pathway
mTOR signaling pathway
MAPK signaling pathway
Insulin signaling pathway
HIF−1 signaling pathway
Cytokine−cytokine receptor interaction
Chemokine signaling pathway
Aminoacyl−tRNA biosynthesis
KEGG Terms
0.04
0.05
0.06
0.07
0.08
0.09
RichFactor
Gene_number
4
6
8
10
12
14
Pvalue
0.075
0.050
0.025

B

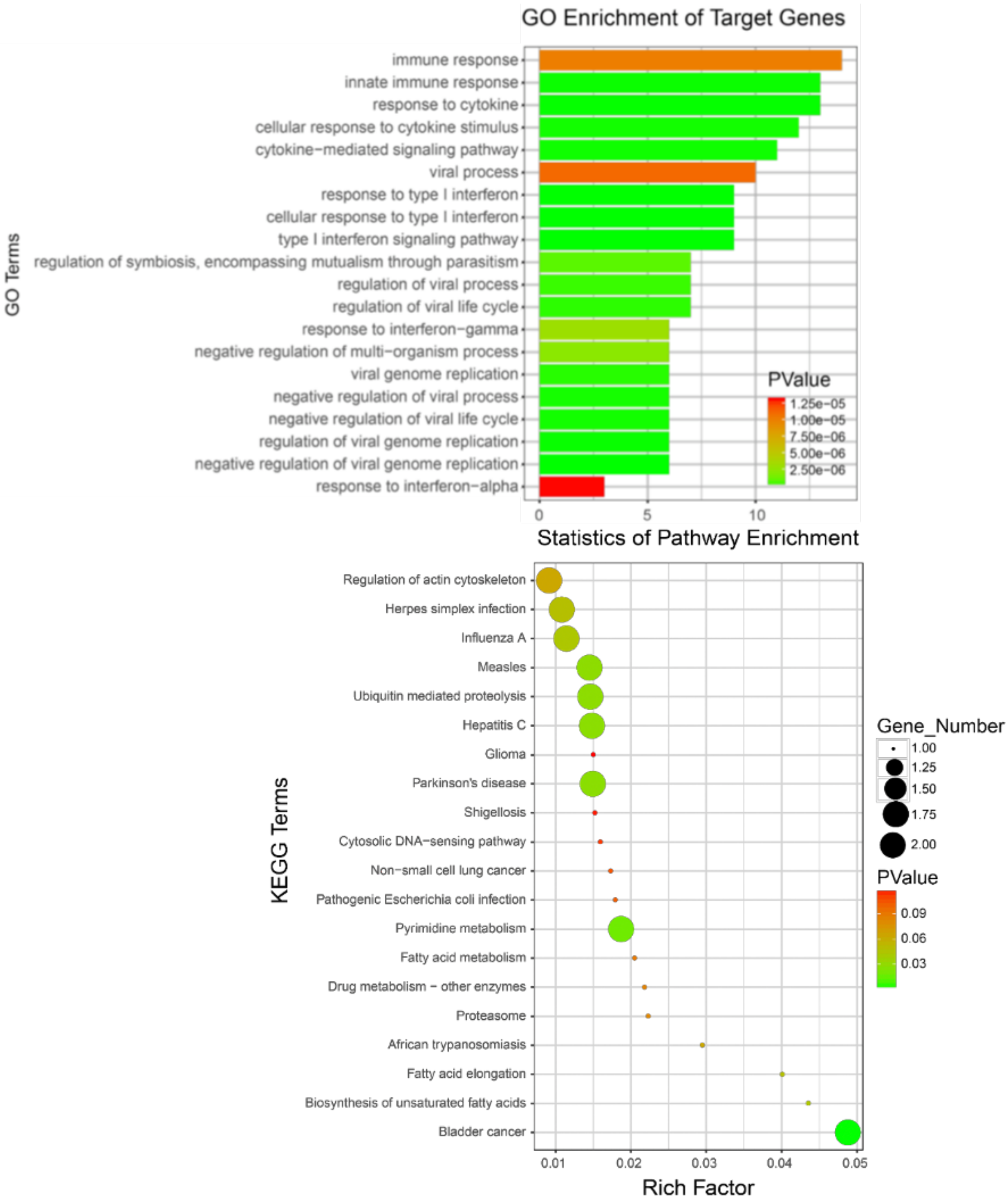

Figure S1. KEGG pathway enrichment and GO analysis of the differential genes regulated by UL23. (A) Used DAVID6.8 to conduct GO analysis and KEGG pathway enrichment analysis on differentially expressed genes in HFF-Vector-IFN-γ group and HFF-UL23-IFN-γ group. (B) Used DAVID6.8 to conduct GO analysis and KEGG pathway enrichment analysis on differentially expressed genes in HCMV-Towne group and HCMV-Towne-ΔUL23 group.

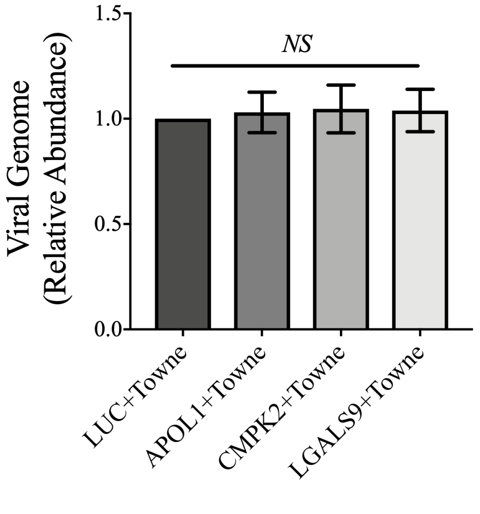

Figure S2. The effect of APOL1, CMPK2, and LGALS9 on HCMV entry into cells. HFF cells were infected with HCMV-Towne at an MOI of 1. Cells were harvested at 4 h and the relative number of viral genomes was determined by qPCR using primers to the UL83 gene region.
